# The implications and consequences of maternal obesity on fetal intrauterine growth restriction

**Published:** 2013-09-25

**Authors:** L Radulescu, O Munteanu, F Popa, M Cirstoiu

**Affiliations:** * Biochemistry Department, “Carol Davila" University of Medicine and Pharmacy, Bucharest; ** "Carol Davila" University of Medicine and Pharmacy, Department of Obstetrics and Gynecology of University Hospital, Bucharest; *** Carol Davila" University of Medicine and Pharmacy, Department of General Surgery, “Sf. Pantelimon" Clinical Emergency Hospital, Bucharest; **** "Carol Davila" University of Medicine and Pharmacy, Department of Obstetrics and Gynecology of University Hospital, Bucharest; Project Manager of “Infrastructure development and translational research in molecular pathology and imaging – MOLIMAGEX"

**Keywords:** obesity, intrauterine growth restriction

## Abstract

** Context.** The prevalence of maternal obesity has been increasing dramatically in the recent years (body mass index ≥ 30 kg/m2). Maternal obesity is associated with an unequivocal increase in maternal and fetal complications of pregnancy and more than that, these complications also extend beyond fetal life in childhood and adulthood.

** Objective.** The aim of this study was to evaluate maternal and neonatal complications at birth associated with maternal obesity.

** Materials and methods.** The study included all women who gave birth between January 1, 2012 and December 31, 2012 at Bucharest University Emergency Hospital. Collected data included information about maternal health (the degree of obesity, associated complications of birth, anemia, and type of birth) and neonatal status (birth weight, gestational age, associated diseases and Apgar score).

** Results.** A higher incidence of IUGR, as well as an increased frequency of infants who needed intensive care after birth, a higher rate of cesarean surgery and a higher frequency of thromboembolic complications were observed in patients with associated obesity.

** Conclusions. ** Complications grow both in number and severity with increasing obesity. Diagnosis of the fetuses with IUGR is important for the monitoring and management of the pregnancy associated with obesity and it involves a close collaboration between obstetrician, family physician and neonatologist.

Abbreviations: IUGR- intrauterine growth restriction

## Background

The prevalence of obesity (defined as body mass index (BMI)> 30 kg/m2) [**1**] is increasing even among women of childbearing age. In the last decade, it has become one of the most common nutritional diseases in the world with the magnitude of a pandemic. According to the WHO report in 2011, it is considered the disease of the XXI century [**2**]. According to a survey conducted in 79 countries, WHO considers that there are 250 million obese people worldwide, of whom an estimated of 22 million are children under the age of 5 years, emphasizing the idea that 50% of the obese children will become obese adults [**3**]. Obesity involves multiple interactions between genetic, social, behavioral, metabolic, cellular and molecular factors leading to changes that result in energy imbalance. Increasing global prevalence of obesity and overweight is due, on one hand, to the decrease in the physical activity and increased sedentariness, and on the other hand to an increased energy intake, particularly in increased density and caloric foods that are rich in fats and sugars. The risk of becoming obese adults of children who developed obesity in the early years is 80% for those with both parents obese and 40% for children with one obese parent [**4**]. 

 According to a study made in Romania in 1980, in the west of the country on a sample of 5,250 children aged 3 months to 16 years, there has been a prevalence of obesity of 14.7%, i.e. 18,6% in infants, 15% to 14.2% preschool and school, with a predominance in women [**5**]. 

 WHO reported a prevalence of overweight in children aged 0-4 years, 6.4% in girls and 5.5% for boys. Data are provided by studies made in the National Nutrition Surveillance Registry 1993-2002 by the Institute of Mother and Child (IMCC) “Rusescu", Bucharest [**6**].

## Material and methods

We analyzed the data of patients who gave birth between January 1st, 2012 and December 31st, 2012, in the Department of Obstetrics and Gynecology of the Bucharest University Emergency Hospital. The inclusion criterion was the diagnosis of maternal obesity without taking into account whether or not the patient was obese before pregnancy. The notion of maternal obesity was defined and classified into grades as indicated by WHO according to BMI as it follows: I degree obesity (BMI=30 to 34.9 kg/m2), II degree obesity with a BMI (35 to 49.9 kg/m2) and III degree obesity with a BMI greater than 40 kg/m2. The definition of obesity was associated with the terms of weight excess and edematous in terms edematous eclamptic pregnancy as they are synonymous in current practice. Maternal anemia was considered at a hemoglobin level <12 g / dL and hematocrit <37. We defined prematurity as the duration of pregnancy of less than 37 weeks, intrauterine growth restriction (IUGR) was defined as newborns weighing less than 2 standard deviations or below 10th percentile corresponding to the average weight for gestational age and macrosomia as a newborn weighing more than 2 standard deviations or above the 90th percentile for the average weight corresponding gestational age. IUGR and fetal macrosomia were diagnosed by ultrasound biometry and postnatal assessment. All neonates were examined thoroughly to rule out major and minor birth defects.

## Results

**Fig. 1:**
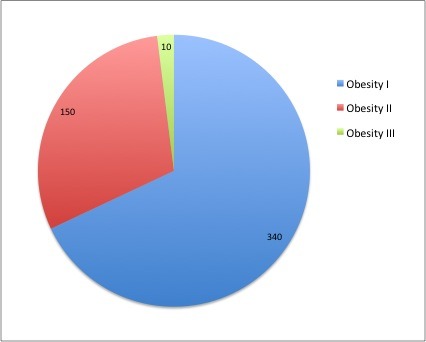
Distribution of patients according to the degree of obesity

Of all the patients who gave birth between January 1st, 2012 - December 31st, 2012 we included in the study 500 of whom 340 patients were included in group I with Ist degree obesity, 150 patients with IInd grade obesity were included in group II and 10 patients with IIIrd degree obesity were included in group III (**Fig. 1**).

 A higher rate of caesarean section was found in the IIIrd group with a frequency of 80% as opposed to a frequency of 42.06% for vaginal delivery in the first group.

 Seven patients gave birth to twins, 4 from group I, 2 in group II and 1 patient in group III.

 From the point of view of intrapartum complications, 20% (2/10) of the patients in group III had an inefficient uterine retraction compared to 5.33% (8/150) in the second and 3.24% (11/340) in first group. 13.33% (20/150) of the patients in group II required labour induction as opposed to 4.41% (15/340) in Group I and 10% (1/10) in Group III. Fetuses extraction was difficult in 30% (3/10) of the patients of the IIIrd group and forceps was used and 8% (12/150) of the IInd group patients had meconium colored amniotic fluid (**Table 1**).

**Table 1:** Number of patients with associated complications according to the obesity degree

Maternal complications	Obesity I	Obesity II	Obesity III
post surgery seroma	42	16	5
postpartum hemorrhage	7	11	3
postpartum endometritis	7	4	2
superficial thrombophlebitis	4	6	4
profound thrombophlebitis	1	1	2
pulmonary thrombembolism	0	1	1
urinary tract infection			
difficult fetal extraction	30	11	2
inefficient uterine retraction	13	9	3
premature rupture of membranes	11	8	2
induction of labour	18	13	1
meconial amniotic fluid	10	12	2
baby blues	12	8	2
lactation deficiency	16	22	3

**Fig. 2:**
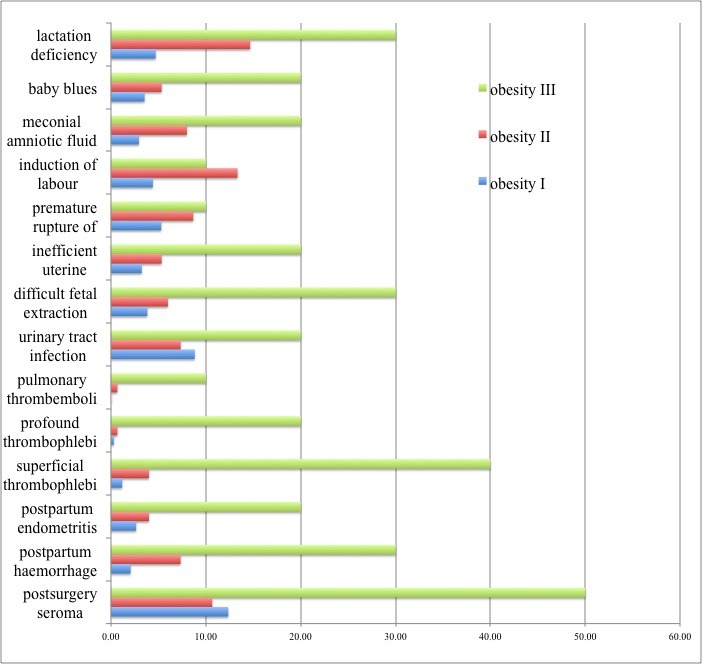
Frequency of maternal complications according to the degrees of obesity

As for the development of postnatal maternal complications, wound seroma was found in 12.35% (42/340) of group I patients, 10.67% (16/150) in group II and 50% (5/10) in patients of group III. Postpartum endometritis was observed in 2.06 % (7/340) of the patients in group I, 2.67% (4/150) in group II and 20% (2/10) in group III. Thromboembolic complications with pulmonary thromboembolism was observed in two of the patients with IIIrd degree obesity, as for superficial thrombophlebitis, 4 out of 10 of the patients in Group III had developed it as opposed to 4% (6/150) of the patients in the group II and 1.18% (4/340) of the patients in Group I. Two of the patients in group III, one in group II and one in group I developed profound thrombophlebitis (**Fig. 2**). 

 In the analyzed group of newborns, a rate of 50% (5/10) of children born by the mothers in group III had intrauterine growth restriction, 26.67% (40/150) in group II and 14.41% (49/340) in group I.

 Approximately 30% of the cases had an Apgar score of 8 to 1 minute in group I and 28.67% had an Apgar score of 9 to 1 minute in group II. We found an increase in value from 1 minute to 5-minute Apgar scores for each group (**Table 2**, **Table 3**).

**Table 2:**
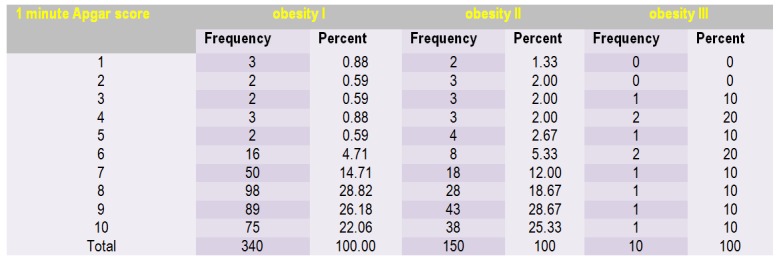
Frequency of 1-minute Apgar score

**Table 3:**
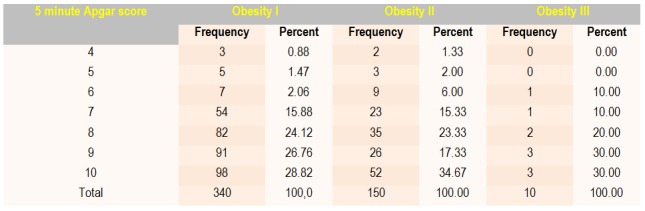
Frequency of 5-minute Apgar score

 Among infants with high birth weight for gestational age, five had birth trauma in group I, 5 in group II and 2 in group III.

 Also, out of the fetal complications, respiratory distress syndrome was found in 20% (2/10) of Group III infants, 6% (9/150) of group II and 3.82% (13/340) of group I and severe hypoxia due to meconium aspiration was found in 20% (2/10) of the infants in group III. In total, out of the 66 infants, the total number of those who were treated throughout 2012 with curiosulf, 27 had obese mothers (**Fig. 4**).

**Fig. 3:**
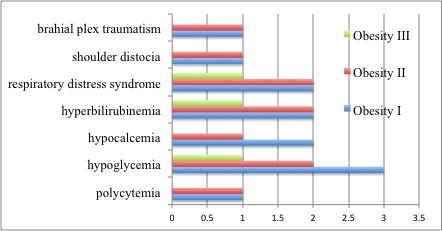
LGA complications divided by maternal obesity degree

**Fig. 4:**
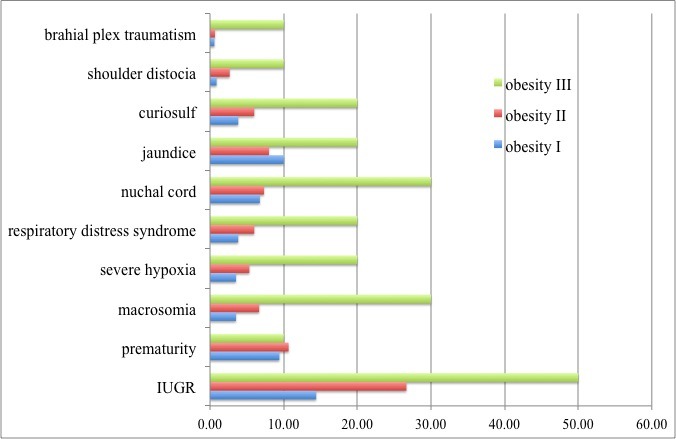
Fetal complications divided according to the maternal obesity degree

## Discussion and conclusions

Complex relationships between maternal metabolic environment of the developing fetus and the potential influence of postnatal life style and environment have complicated the efforts to study the effects of maternal programming of overeating in humans.

A proposal to explain the link between maternal obesity and child obesity is the “overeating hypothesis". It states that hyperglycemia, increased levels of free fatty acids and amino acids cause permanent changes in appetite control, neuroendocrine functioning and/ or energy metabolism in the developing fetus, leading to the emergence adiposity risk (with risks of metabolic and cardiovascular disease) later in life [**7**].

 It is well known that the events in the womb have long-term influences on the risk of disease later in life. This phenomenon, known as “early life programming", has been extensively studied in relation to low birth weight, with the adjustment of the developing fetus in the womb (for example due to maternal malnutrition), in order to maximize the immediate chance of survival [**8**]. These adaptations include permanent changes in structural axes, physiological and hormonal phenomenon of down-regulation of growth resulting low birth weight [**9**].

 Maternal obesity and overeating are now recognized as “programming factors". 

 Intrauterine growth restriction (IUGR) is an important public health problem in both industrialized and developing countries, leading to perinatal morbidity and long-term sequelae and mortality. The correct identification of IUGR has a great importance as the low weight of the newborn determines the specific conduct, surveillance as well as antenatal and postnatal care.

 Obesity during pregnancy increases the risk for a number of complications for both the mother and child.

 The recommendation for weight gain during pregnancy is of 11.2 - 15.9 kg (0.5-2.0 kg for the first trimester and 0.35 - 0.50 kg per week for the second and third trimesters) (**Table 4**) [**10**].

**Table 4:** WHO Recommendations for the weight gained during pregnancy

Body Mass Index (BMI) (weight kg/height m2)	Recommended weight gain
18.5–24.9 kg/m2 (normal weight)	11.2–15.9 kg
25–29.9 kg/m2 (overweight)	6.8–11.2 kg
> 30 kg/m2 (obese)	6.8 kg

 Although there are strict recommendations for overweight and obese pregnant women to keep their weight gain to a minimum, women who are overweight before pregnancy are more likely to exceed the recommendations and have a higher risk of complications [**11**]. 

 Maternal obesity is associated with an increased risk of perinatal mortality and the occurrence of genetic disorders. The most common complications are the death of the fetus in utero, genetic disorders, macrosomia and intrauterine growth restriction [**12**].

 Fetal death is a dramatic result of any pregnancy, especially when it occurs late in the pregnancy. An increase of up to five times the risk of intrauterine death and increased infant mortality in obese women was recorded in some studies [**13**]. Also there seems to be a correlation between maternal BMI and infant mortality [**14**].

 An explanation of the increased incidence of congenital anomalies in fetuses of obese women could be represented by the difficulties of interpretation of blood serum indices and failure to display fetal anatomy on the ultrasound. However, there is data to justify a real association between maternal obesity and genetic disorders. Specifically, fetuses of obese mothers have a higher risk of developing neural tube defects such as spina bifida, heart defects and abdominal wall defects such as omphalocele. These abnormalities are more common in children with mothers with diabetes mellitus type 2 and folic acid deficiency, disorders that often coexist with obesity.

 Numerous studies have established the association between maternal obesity and insulin resistance weight before pregnancy and fetal health, concluding that they affect fetal growth [**15**].

 Obesity and insulin resistance modify the placental function, in the last weeks of pregnancy, increasing the availability of glucose, fatty acids and amino acids to the fetus [**16**]. The induced fetal hyperglycemia as a result of maternal hyperglycemia leads to hypertrophy/ hyperplasia of the pancreas and fetal hyperinsulinemia. Insulin has a direct effect on cell division, resulting in macrosomia. Therefore, women with diabetes have an increased risk of macrosomia. Given that the prevalence of obesity is about ten times larger than gestational diabetes, it is obvious that the lifestyle of the mother exerts a great influence on the incidence of fetal macrosomia [**17**].

 A common etiology of intrauterine growth restriction is the placental pathology including placental insufficiency, anatomical abnormalities, such as corioamnionitis, hemangiomas, placental tumors, single umbilical artery, placental abruption and placenta praevia.

 Fetal etiopathogenic factors are genetic defects, chromosomal and cardiovascular abnormalities, congenital infections and metabolic diseases [**18**].

 Maternal obesity is also associated with a significantly increased risk of low Apgar score at birth [**19**].

 In a Swedish study conducted on a sample of 189,783 children, a higher maternal BMI was associated with a higher risk of asthma; children with obese mothers are more likely to require medication and hospitalization for asthma at age 8-10 [**20**]. Also, a study conducted on a sample of 6945 Finnish adolescents found that a high prepregnancy BMI indicates an occurrence of wheezing and asthma in children aged 15-16 years [**21**].

 Maternal obesity has also been linked to impaired brain development and behavioral changes in children. A study involving a total of 1,004 children found that 67% of obese mothers were more likely to have a child with an autism spectrum disorder, diagnosed with standardized assessments, and twice as likely to have a child with a developmental delay [**22,23**]. Another study conducted on a sample of 1714 children aged 5 years has shown that obese patients with obese mothers are more likely to develop symptoms of attention deficit hyperactivity disorder (ADHD), lack of concentration and difficulty regulatory emotionality as reported by kindergarten teachers and mothers using a list of DSM-IV symptom-derived [**24,25**]. Further studies are needed to determine whether these possible negative effects of maternal obesity on brain function of children persist in adult life.

 In any human study, although factors related to lifestyle, such as the current level of obesity, behavior, activity and diet are often considered as confounding factors in the statistical analysis, it is almost impossible to separate pre- and postnatal influences on children's outcomes [**26-28**]. Also, common maternal genes that influence the risk of obesity of children should be considered.

 Studies in siblings were used as an attempt to separate the intrauterine events from environment and genetic factors, and a recent study showed an independent influence of maternal obesity and weight gain during pregnancy on children, especially among women obese [**29**].

 The numerous studies conducted on different populations emphasize the association between intrauterine growth restriction and peri and postnatal evolution as differing depending on fetal sex. And, although intrauterine programming mechanisms are still unclear and the involvement of other factors and results of the studies are controversial, it seems that the female gender is more likely to develop intrauterine growth restriction [**30**].

 Perhaps, more evidence of maternal obesity on children's programming comes from a study that used a group of mothers who had undergone surgery for obesity [**31**]. The authors were able to observe the long-term effects. Children born before their mothers had undergone biliopancreatic diversion (BPD) for obesity had significantly higher body weights at 12 and at 21 to 25 years than children born after surgery. Thus it supports the hypothesis that obesity has long-term influences on children's weight and BMI independent of genetic, environmental and lifestyle. However, it is likely that dietary changes made by these mothers have influenced postoperative diet and lifestyle of children born after surgery.

 In conclusion, maternal obesity is a serious health risk for the fetus, the impact increasing according to the degree of obesity. A non-balanced diet during pregnancy not only contributes to the abnormal development of the fetus and the subsequent increase in the neonatal morbidity and mortality, but also to increased morbidity during childhood, adolescence and adulthood. Due to the difficulties in identifying, monitoring these fetuses, prenatal as well as postnatal, and because of the increased perinatal mortality and morbidity associated intrauterine growth restriction (IUGR) remains a serious multidisciplinary problem.

 A systematic effort to reduce weight is imperative to avoid transmission of obesity from generation to generation. Achieving this goal will most likely result in a sharp decrease in fetal and neonatal morbidity and mortality and improve outcomes followers and future pregnancies.

Disclosure: None of the authors have a conflict of interest.

 Acknowledgement: 

 This paper is supported by the Sectoral Operational Programme Human Resources Development (SOP HRD) 2007-2013, financed from the European Social Fund and by the Romanian Government under the contract number POSDRU/107/1.5/S/82839.
